# Identification of prognosis-related genes and construction of multi-regulatory networks in pancreatic cancer microenvironment by bioinformatics analysis

**DOI:** 10.1186/s12935-020-01426-1

**Published:** 2020-07-25

**Authors:** Tong Li, Qiaofei Liu, Ronghua Zhang, Quan Liao, Yupei Zhao

**Affiliations:** Department of General Surgery, Peking Union Medical College Hospital, Chinese Academy of Medical Sciences & Peking Union Medical College, Beijing, 100730 China

**Keywords:** TCGA, Differentially expressed genes, Multi-regulatory network, Pancreatic cancer, Tumor microenvironment

## Abstract

**Background:**

As one of the most lethal cancers, pancreatic cancer has been characterized by abundant supportive tumor-stromal cell microenvironment. Although the advent of tumor-targeted immune checkpoint blockers has brought light to patients with other cancers, its clinical efficacy in pancreatic cancer has been greatly limited due to the protective stroma. Thus, it is urgent to find potential new targets and establish multi-regulatory networks to predict patient prognosis and improve treatment.

**Methods:**

We followed a strategy based on mining the Cancer Genome Atlas (TCGA) database and ESTIMATE algorithm to obtain the immune scores and stromal scores. Differentially expressed genes (DEGs) associated with poor overall survival of pancreatic cancer were screened from a TCGA cohort. By comparing global gene expression with high vs. low immune scores and subsequent Kaplan–Meier analysis, DEGs that significantly correlate with poor overall survival of pancreatic cancer in TCGA cohort were extracted. After constructing the protein–protein interaction network using STRING and limiting the genes within the above DEGs, we utilized RAID 2.0, TRRUST v2 database and degree and betweenness analysis to obtain non-coding RNA (ncRNA)-pivotal nodes and TF-pivotal nodes. Finally, multi-regulatory networks have been constructed and pivotal drugs with potential benefit for pancreatic cancer patients were obtained by screening in the DrugBank.

**Results:**

In this study, we obtained 246 DEGs that significantly correlate with poor overall survival of pancreatic cancer in the TCGA cohort. With the advent of 38 ncRNA-pivotal nodes and 7 TF-pivotal nodes, the multi-factor regulatory networks were constructed based on the above pivotal nodes. Prognosis-related genes and factors such as HCAR3, PPY, RFWD2, WSPAR and Amcinonide were screened and investigated.

**Conclusion:**

The multi-regulatory networks constructed in this study are not only beneficial to improve treatment and evaluate patient prognosis with pancreatic cancer, but also favorable for implementing early diagnosis and personalized treatment. It is suggested that these factors may play an essential role in the progression of pancreatic cancer.

## Background

Pancreatic cancer currently is one of the top three leading causes of cancer-related death in the United States and its death toll is rising dramatically [1]. The only chance of cure for pancreatic cancer patients is surgical resection [2]. For decades, despite the concerted efforts, the five-year survival rate remains dismal for all stages combined [1]. The lack of early detection tests and recognizable symptoms or signs result in late diagnoses [3]. The advent of various systemic therapies has just provided limited efficacy for pancreatic cancer patients to date [4, 5], which may be due to the abundance of tumor stromal content [6, 7].

The pancreatic cancer stroma, which has aptly been termed the tumor microenvironment (TME), is the cellular milieu where the tumor is located. The stroma occupies the majority of the tumor mass and is heterogeneously comprised of dynamic assortment of non-neoplastic cells and extracellular matrix components, including fibroblastic, vascular and immune cells, cytokines and growth factors [8, 9]. The influences of the stroma in pancreatic cancer are as manifold as its components [7]. In the pancreatic TME, immune cells and stromal cells are the two main types of non-neoplastic components and have been proposed to be of value for the diagnosis and prognosis of tumors [10–13].

The comprehensive understanding of immune cells and stromal cells in pancreatic cancer tissues may shed light on the tumor pathophysiology and help with developing wholesome prognostic and predictive models [8, 14]. Thus, it is important to evaluate immune scoring and matrix scoring to investigate the composition of stromal cells and immune cells and evaluate tumor purity [12, 14, 15]. Algorithms [15, 16], such as ESTIMATE (Estimation of Stromal and Immune cells in Malignant Tumor tissues using Expression data), have been developed to predict tumor purity and the infiltration of non-tumor cells by calculating immune and stromal scores by utilizing gene expression data from the Cancer Genome Atlas (TCGA) database [17]. In this ESTIMATE algorithm, we analyzed immune and stromal cells of their specific genes expression characteristics to obtain immune and stromal scores and predict invasion of non-tumor cells [18]. Subsequent researches have shown the effectiveness of applying the ESTIMATE algorithm in various tumors [17, 19–21], although its utility on pancreatic cancer have not been fully revealed.

The present study, for the first time in decades, by utilizing both TCGA pancreatic cancer data and immune scores from ESTIMATE algorithm [15], we obtained a list of genes, non-coding RNAs (ncRNAs) and transcription factors (TFs) that were associated with microenvironment and could predict poor outcomes in pancreatic cancer patients, along with possible effective drugs. Essentially, we have validated the correlation in the International Cancer Genome Consortium (ICGC) database, which is a different pancreatic cancer cohort.

## Methods

### Acquisition of gene expression data of pancreatic cancer from TCGA and ICGC database

Gene expression profile of pancreatic cancer patients and their clinical characteristics were extracted from the TCGA database (https://tcga-data.nci.nih.gov/tcga). Use of the TCGA data adhered to TCGA publication guidelines and policies. By utilizing the ESTIMATE algorithm to the downloaded data, we obtained the immune and stromal scores [22]. In addition, gene expression profiles and clinical data of survival and outcomes for pancreatic cancer patients were extracted from the ICGC database (https://icgc.org) for validation. These normalized data were used for LIMMA analysis and survival analysis. The summary of the clinical information of pancreatic cancer cohorts from TCGA is presented in Additional file 1: Table S1.

### Identification of differentially expressed genes (DEGs) and functional enrichment analysis

**Fig. 1:**
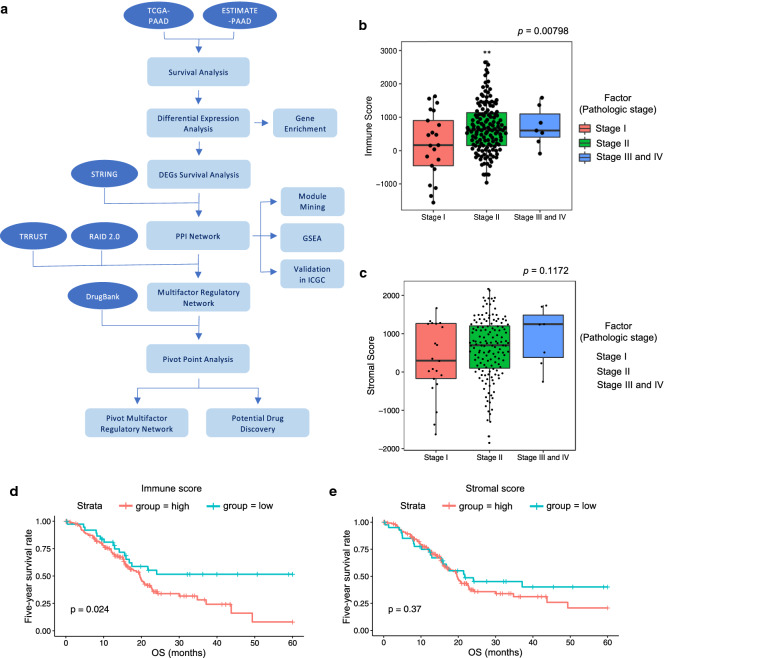
Immune scores and stromal scores are associated with pathologic stages and overall survival of pancreatic patients. **a** Work flow of the present study. **b** Distribution of immune scores of different pathologic stages. Box‐plot shows that there is significant association between pathologic stages and the level of immune scores (n = 177, p = 0.00798). **c** Distribution of stromal scores of different pathologic stages. Box‐plot shows that there is no significant association between pathologic stages and the level of stromal scores (n = 177, p = 0.1172). **d** TCGA cohort samples were divided into two groups based on their immune scores: the 139 cases with immune scores higher than 0 and the 38 cases with immune scores lower than 0. As shown in the Kaplan‐Meier survival curve, median survival of the low score group is longer than high score group (p = 0.024 in log-rank test). **e** Similarly, TCGA cohort samples were divided into two groups based on their stromal scores: the 135 cases with stromal scores higher than 0 and the 42 cases lower than 0. The median survival of the low score group is longer than the high score group. However, it is not statistically different as indicated by the p = 0.37 in log-rank test

The inclusion criteria for identification of DEGs were set as fold change (FC) > 2 and adjusted *p* value < 0.05. The DEGs were chosen for heatmaps and clustering by means of pheatmap and an open source web tool ClustVis [23]. The KEGG pathway enrichment analysis for identified DEGs was conducted using a web server named KOBAS (version 3.0) (KEGG Orthology Based Annotation System) [24] online database server (http://kobas.cbi.pku.edu.cn/) with the thresholds of p-value < 0.05. The flow chart was shown in Fig. 1a.

### Construction of PPI network

STRING (version 10.5) tool was utilized to establish the protein–protein interaction (PPI) network [25]. Subsequently, the visualization and analyse was achieved via Cytoscape (version 3.6.1, http://www.cytoscape.org/) [26]. To locate densely populated regions based on topology, we used the plug‐in Molecular COmplex DEtection (MCODE) to filtrate significant modules of the PPI network (the parameters were set to default).

### Overall survival curve

Based on the gene signature of multiple survival-associated DEGs, Kaplan–Meier plots were plotted to elaborate the association between patients’ 5-year overall survival and DEGs expression levels. The relationship was tested by log-rank test.

### Degree and betweenness analysis of the PPI interaction network with module identification and pivot ncRNA recognition

The network was analyzed by using Network Analyzer, a Cytoscape plug-in, based on topological parameters such as degree and betweenness [27]. In summary, degree illustrates the amount of edges linked to a specific node, while betweenness determines the sum of nonredundant shortest paths passing through a specific node/edge, identifying the network bottleneck [28]. The PPI network was clustered with the ClusterONE algorithms (in Cytoscape) so as to determine functional modules [29]. Based on the PPI network, ClusterONE could detect the protein complex module by altering the most valuable incident and boundary nodes time and again to locally optimize the cohesiveness measure of cluster quality metrics [29]. The pivot ncRNAs that significantly modulate distinct modules and sub-networks are identified by a hypergeometric test [30].

### Analysis of targeting drugs

The DrugBank online database (https://www.drugbank.ca/) [31, 32] is contained of biochemical and pharmacological information of any kinds of drugs. In this study, we filtered the data from DrugBank to determine the drug target information of module genes in PPI network and subsequently built drug-target gene interaction networks.

### Timer 2.0 database analysis

To analyze the association of gene expression and immune infiltration level of immune cells including CD8+T cell, CD4+T cell, Treg cell, B cell, neutrophil, macrophage, dendritic cell, natural killer cell and monocyte, the online public resource Tumor Immune Estimation Resource 2.0 (TIMER 2.0; http://timer.cistrome.org/) was utilized [33, 34].

## Results

### Immune and stromal scores are associated with pathologic stages and overall survival of pancreatic cancer patients

The gene expression profiles and clinical information of all 177 pancreatic cancer patients (with initial pathologic diagnosis) were downloaded in TCGA cohort. We included all the pancreatic cancer cases with complete gene expression data and clinical information in the TCGA in our analysis. There clinicopathologic information was listed in Additional file 1: Table S1. According to the clinical information recorded in the TCGA database, we divided all pancreatic cancer cases into Stage I (21/177, 11.9%), Stage II (146/177, 82.5%) and Stage III and IV (7/177, 4.0%) subgroups based on the overall stage of cancer. Their immune and stromal scores were obtained from the ESTIMATE website. The numerical distribution of the two scores was shown in Fig. 1B. In accordance with ESTIMATE algorithm, immune scores were distributed between − 1559.87 and 3037.78 and stromal scores ranged from − 1843.32 to 2179.19, respectively. As shown in Fig. 1b, the pancreatic cancer cases at Stage II subgroup had the highest average immune score (p < 0.01), followed by Stage III and IV, while the Stage I samples had the lowest immune score. Similarly, the rank order of stromal scores across pancreatic cancer subgroups from highest to lowest was Stage III and IV > Stage II > Stage I, although the differences were not statistically significant (Fig. 1c), indicating that immune scores are meaningful in the correlation of subgroup classification.

To find out the potential correlation of immune and/or stromal scores with overall survival, we divided the 177 pancreatic cancer cases into high vs. low score groups (using zero as the cut-off point) and performed survival analysis using clinical follow-up data for each set of samples. Kaplan–Meier survival curves indicated that 5-year survival rate of cases with low immune scores was longer (Fig. 1d log-rank p = 0.024). Consistently, although not statistically significant, longer 5-year overall survival were observed in cases with lower, compared to higher, stromal scores (Fig. 1e, log-rank p = 0.37).

### Comparison of gene expression profile with immune and stromal scores in pancreatic cancer

**Fig. 2:**
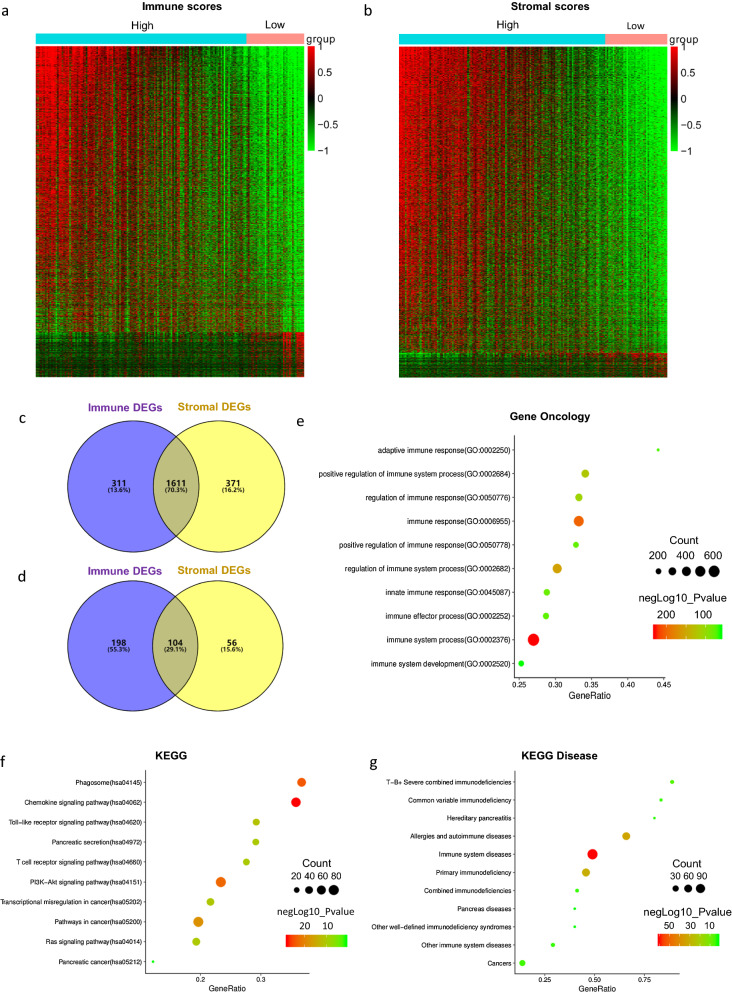
Comparison of gene expression profile with immune scores stromal scores in pancreatic cancer Heatmaps were drawn based on the methods of average linkage and Pearson distance measurement. Genes with higher expression are shown in red while lower expression genes are shown in green. **a** Heatmap of the DEGs of immune scores higher than 0 vs. immune scores lower than 0 (p < 0.05, fold change > 2). **b** Heatmap of the DEGs of stromal scores higher than 0 vs. immune scores lower than 0 (p < 0.05, fold change > 2). **c**, **d** Venn diagrams showing the number of commonly upregulated (**c**) or downregulated (**d**) DEGs in immune and stromal score groups. Top 10 terms of GO (**e**), KEGG pathway (**f**) and KEGG disease (**g**) were acquired from KOBAS 3.0 annotation and enrichment tool. p < 0.05

To explore the correlation of global gene expression profiles with immune and/or stromal scores, we analyzed the RNAseq - HTSeq - FPKM data of all 177 TCGA pancreatic cancer cohorts. As the heatmaps shown in Fig. 2a and b, there were evident gene expression profiles between cases of high vs. low immune/stromal score groups. According to the immune scores, a total of 2224 DEGs were screened with 1922 upregulated and 302 downregulated genes in high vs. low score groups (FC > 2, p < 0.05). When it comes to stromal scores, there were 1982 up-regulated genes and 160 down-regulated genes screened in high vs. low score groups (FC > 2, p < 0.05). In addition, the Venn diagrams in Fig. 2c, d showed 1611 commonly up-regulated genes and 104 commonly down-regulated genes in the high score groups. There is a certain degree of overlap between the DEGs in the high-scoring and low-scoring groups of the immune score and the stromal score, especially in the up-regulation group. Among the differentially up-regulated genes, 1611 genes were shared between immune and stromal score groups, accounting for 70.3% of the genes. While the 104 common down-regulated genes accounted for 29.1%. It is worth mentioning that only immune scores were significantly correlated with 5-year overall survival of patients. Therefore, we were determined to focus on the DEGs from immune score groups for all the following analyses in this manuscript.

Then, by using KOBAS 3.0, functional enrichment analysis of all upregulated 2224 genes in high-immune scores group were carried out in order to sketch the possible effect of the DEGs between the two immune score groups (high and low). The DEGs we obtained in the GO annotation and relevant pathways were enriched in the KEGG database. As shown in Fig. 2e–g, numerous genes were associated with tumor microenvironment or immune function.

### Correlation of expression of individual DEGs in overall survival

To determine the separate effects of the 2224 DEGs on 5-year overall survival, Kaplan–Meier survival curves were generated, of which 246 genes in total were identified to be significantly associated with poor overall survival prediction (log-rank p < 0.05, Additional file 1: Figure S1 shows designated genes).

### Protein-protein interactions among genes of prognostic value

**Fig. 3:**
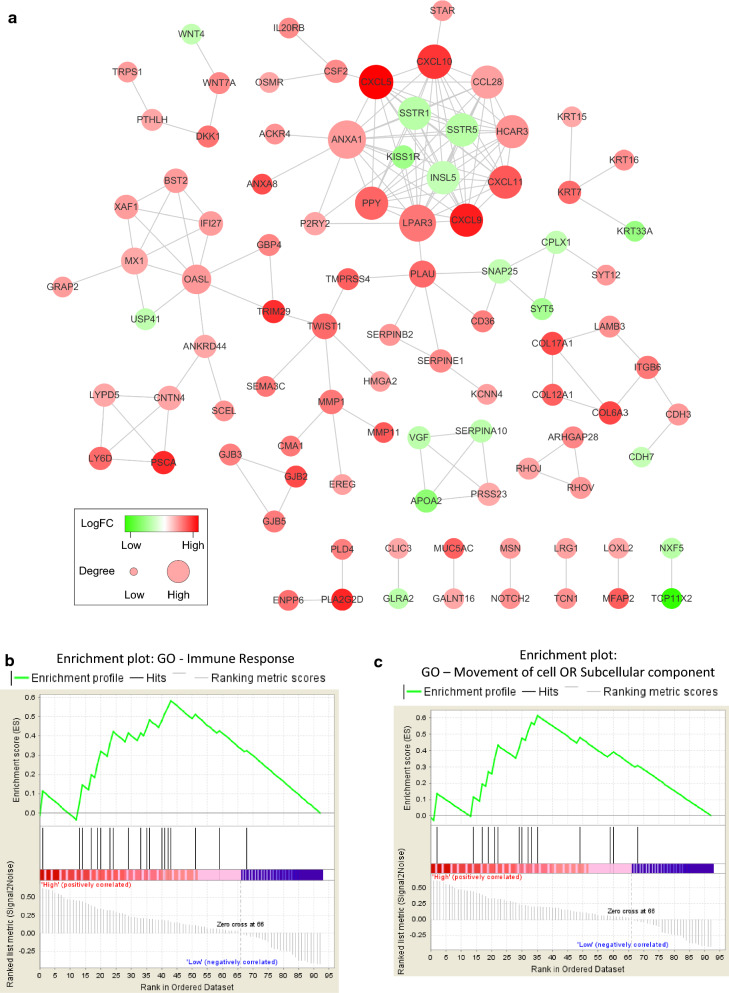
PPI networks among genes of prognostic value and functional enrichment analysis. **a**PPI networks among DEGs (93 nodes) that are significantly associated with 5-year overall survival. The color of a node in the network reflects the log (FC) value of the Z score of gene expression. The size of a node indicates the number of interacting proteins with the designated protein. **b**, **c** Results of GSEA analysis. GSEA analysis was performed for the 93 DEGs to further screen the significant GO between the higher immune scores group and lower immune scores group. GSEA, gene set enrichment analysis

To investigate the interactions among the above DEGs, we utilized the STRING tool and yeasted protein–protein interaction (PPI) networks. By limiting the genes with interactions to be only included in these 246 DEGs, we established a PPI network that are significantly associated with 5-year overall survival using Cytoscape software. As suggested by Fig. 3a, 93 nodes and 158 edges composed the network. As former studies reported [35–37], there is biological significance in PPI networks (nodes = proteins; edges = interactions between proteins). It is generally believed that important proteins often cooperate with many other proteins (such as transcription factors). In other words, nodes (proteins) with larger node degrees (more interactions and cooperations) in the network are thus more important for further study.

### Functional enrichment analysis for DEGs of prognostic value

To further clarify the main biological functions of the 93 screened DEGs, we performed functional enrichment clustering analysis of the 93 gene nodes using the gene set enrichment analysis (GSEA) method (p < 0.05, FDR < 0.25). As shown in Fig. 3b and c, these genes showed strong association with biologically significant processes such as immune response, cell and subcellular composition and movement of cell or subcellular component, which is consistent with the result of PPI network analysis.

### Module mining of PPI networks

**Fig. 4:**
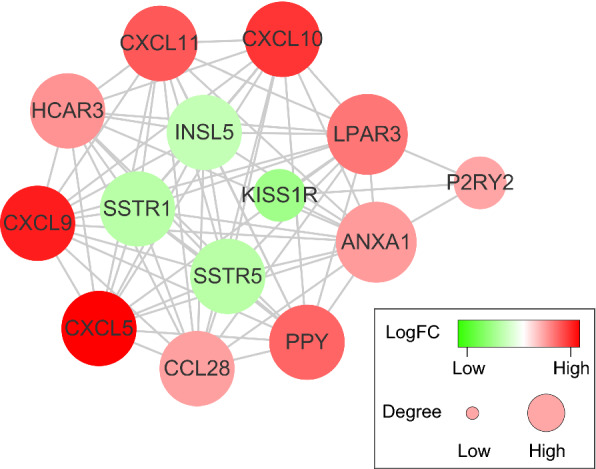
Module mining of PPI networks Results of algorithms from Cytoscape. The PPI network data from STRING was further analyzed by Cytoscape. Circles and lines represent genes and the interaction of proteins between genes, respectively. The color of a node in the network reflects the log (FC) value of the Z score of gene expression. The size of a node indicates the number of interacting proteins with the designated protein

To perform the module mining on the previously mentioned PPI networks, we obtained a total of 7 modules using the MCODE tool in Cytoscape. It is worth mentioning that one module contained more than 10 nodes (Fig. 4). CXCL5, which have been reported to recruit and activate leukocytes and play a role in cancer progression, was also indicated to be one of the most key genes in the PPI network. The genes in the module, including CXCL9, CXCL10, CXCL11, PPY, LPAR3, HCAR3 and so on, were mainly associated with the prognosis of pancreatic cancer or other tumos as listed according to each node degree in Table 1.

**Table 1:** Module mining results of genes associated with the prognosis of pancreatic cancer or other cancers

Degree	Gene	logFC	Reference
13	ANXA1	1.172288	PMID:25854353, PMID:27412958
13	LPAR3	1.616316	PMID:22876164, PMID:26440309
11	SSTR1	− 1.20245	PMID:15706439, PMID:18823376
11	SSTR5	− 1.21848	PMID:15706439, PMID:26474434
11	CXCL5	2.994452	PMID:21356384
11	INSL5	− 1.03296	PMID:25514935
11	CXCL10	2.382618	PMID:25415223, PMID:26423423
11	CXCL11	1.970112	PMID:30747226
11	CCL28	1.114131	PMID:28092365
11	CXCL9	2.672843	PMID:30685120, PMID:23394575
11	HCAR3	1.282322	
11	PPY	1.816823	
3	KISS1R	− 1.62753	PMID:23969598, PMID:30123188
3	P2RY2	1.056005	PMID:30420446

### Validation in the ICGC database

To validate the prognostic significance of the 93 prognosis-related genes identified from the TCGA cohort, we downloaded and analyzed gene expression data and its corresponding clinical information of a cohort of 95 pancreatic cancer cases from ICGC. It showed that this set of data contained 79 of 93 prognostically significant genes and their prognostic values in pancreatic cancer were visualized by Kaplan–Meier survival curves. A total of 26 genes were found to be significantly associated with poor prognosis (Additional file 1: Figure S1), while five of them, including SYT12, GJB5, RHOJ, GJB3 and IFI27, were of particular interest as they have not been previously associated with poor outcomes in pancreatic cancer cases.

### Degree and betweenness analysis with prognosis-related DEGs and multi-regulatory network construction

By using RAID 2.0 and TRRUST v2 database respectively, we investigate the interaction between human ncRNAs along with TFs and the 93 prognosis-related genes. We obtained 8437 out of 1,954,911 pairs of ncRNAs with genes and 179 out of 8427 pairs of TFs with genes. These interaction pairs were imported into Cytoscape software to build a multi-regulatory network with 2228 nodes and 8616 edges, including 2021 miRNA nodes, 3 lncRNA nodes, 89 TF (transcription factor) nodes and 91 mRNA nodes. In order to obtain more accurate and significant results, we used the degree and betweenness analysis method to further screen factors including ncRNAs, TFs and drugs that have significant regulatory effects on prognosis-related DEGs. Here, a pivotal node was defined as having interactions with at least two prognosis-related genes and the hypergeometric p value was less than 0.05.

### ncRNAs regulate genes associated with significant prognosis

To explore ncRNAs that can significantly regulate prognosis-related mRNAs, we screened the RAID 2.0 database for pivotal ncRNA nodes. By using degree and betweenness analysis, we obtained a total of 42 ncRNA-pivotal nodes, including 39 miRNA nodes and 3 lncRNA nodes. The 10 most hypergeometrically significant ncRNAs, including WSPAR, has-miR-4425, has-miR-4536-5p, has-miR-410-5p, has-miR-8064, has-miR-6871-5p, has-miR-4767, has-miR-608, has-miR-4481 and has-miR-3605-3p, were listed in Additional file 1: Figure S2 (in which the expression of has-miR-4481 was not reported in pancreatic cancer).

### Transcription factors (TFs) regulate genes associated with significant prognosis

To explore TFs that can significantly regulate prognosis-related genes, we screened the TRRUST v2 database for pivotal TF nodes. Accordingly, we put nodes that interact with at least two prognosis-related genes (hypergeometric p < 0.05) into the candidate TF node set. Since we only found one TF, RFWD2, based on the hypergeometric p value, we loosened the p value cut-off to 0.1 and obtained 7 TF-pivotal nodes as listed in Additional file 1: Figure S3. Subsequently, we analyzed the association of the expression level of RFWD2 and immune cell infiltration by utilizing the TIMER 2.0 database (Additional file 1: Figure S4).

### Construction of a multi-factor regulatory network based on the pivot nodes

Next, we pruned the multi-regulatory network constructed above based on the obtained ncRNA nodes and TF nodes and removed the rest of the non-pivot nodes. Since there were four miRNAs, including hsa-miR-3978, hsa-miR-4276, hsa-miR-4481 and hsa-miR-4535, whose expressions were not found in pancreatic cancer, thus, the final network included 119 nodes and 241 edges, including 7 TFs, 3 lncRNAs and 35 miRNAs.

### Explore drugs that have a therapeutic effect on pancreatic cancer

To explore more effective drugs that can significantly regulate prognosis-related genes, we screened the DrugBank database for pivotal interaction nodes. Similarly, a pivotal node is defined as having interactions with at least two prognosis-related genes and the hypergeometric p value was less than 0.05. A total of 17 drug-pivot nodes were obtained (hypergeometric p < 0.05). The 10 most significant drugs including Amcinonide and Octreotide were listed in Table 2.

**Table 2:** Top 10 pivotal drugs that have potential therapeutic effects on pancreatic cancer

Drug-pivot	p value
Fibrinolysin	0.000526165
Urokinase	0.00126686
Pasireotide	0.003062761
Octreotide	0.003062761
Lutetium Lu 177 dotatate [PMID:22388631, PMID:28076709]	0.005028043
Tenecteplase	0.025271353
Amcinonide	0.045593349
Botulinum Toxin Type A	0.045593349
HspE7 [PMID:19225922, PMID:11189443]	0.045593349
Lanreotide [PMID:26614375, PMID:25060168]	0.045593349

## Discussion

Recently, increasing evidence has proved that tumor microenvironment plays an essential role in the process of pancreatic cancer [38–41]. Immune cells and stromal cells, the two main types of non-neoplastic components, have been reported to accelerate tumor immune escape and progression [42, 43]. More and more studies have revealed that stroma not only served as a physical barrier to drug delivery and facilitated chemotherapy resistance, but also supported tumor growth and metastasis [44]. Although gemcitabine is presently the standard option in the treatment of pancreatic cancer, stromal components including tumor-associated macrophages have been proven essential in delivering gemcitabine resistance in pancreatic cancer cells [45]. Thus, investigating the mechanism of tumor microenvironment and identifying stromal components could impel a deeper understanding and contribute to a better prognosis of pancreatic cancer patients in clinical practice.

In the present study, we identified numerous tumor microenvironment related DEGs which contribute to pancreatic cancer overall survival in the TCGA database. Especially, by comparing global gene expression in abundant cases with high vs. low immune scores, we obtained 246 genes that significantly correlate with poor overall survival in TCGA pancreatic cancer training cohort. By constructing PPI network and limit the genes only in the 246 DEGs, we obtained 93 gene nodes, in which 79 of them were validated in ICGC pancreatic cancer cohort. The subsequent GSEA method confirmed their strong association with biologically significant processes including immune response and cell and subcellular composition. The module mining further assured their association with the prognosis of pancreatic cancer and beyond. Afterwards, by manipulating RAID 2.0, TRRUST v2 database and pivotal point analysis, we obtained 38 ncRNA-pivotal nodes (with 4 unexpressed miRNAs excluded) and 7 TF-pivotal nodes that interacted with the 93 prognosis-related DEGs. Then, the succeeding multi-factor regulatory network construction was achieved based on the screened pivotal nodes. By utilizing the TIMER 2.0 database, we validated the significant correlation of RFWD2 expression and immune infiltration including CD8+T cell, CD4+T cell, B cell, macrophage and natural killer cell. Ultimately, by screening in the DrugBank, we obtained 17 pivotal drugs that were potentially benefit for pancreatic cancer patients.

As illustrated in Fig. 4 and Table 1, of the 14 genes identified, 12 genes (e.g., ANXA1, LPAR3, SSTR1, CXCL5, KISS1R) have been reported to be involved in pancreatic cancer pathogenesis or significant in predicting overall survival, indicating that the big data-based analyses in the current work using TCGA training cohort and ICGC validation cohort have prognostic values. While the remaining two genes, HCAR3 (Hydroxy-Carboxylic Acid Receptor 3) and PPY (Pancreatic Polypeptide), have not previously been linked with pancreatic cancer prognosis yet, this study provides a theoretical basis for investigating their potential role in pancreatic cancer.

Recently, the explosion of studies into ncRNA have provided evidence of their key regulatory roles in shaping oncogenic activity in pancreatic cancer [46–48]. Subsequently, many cancer-focused clinical trials involving ncRNAs as novel biomarkers have begun [49–51]. In our present work, the top 10 ncRNAs with significant association with pancreatic cancer prognosis were listed in Additional file 1: Figure S2, including WSPAR (lncRNA T-Cell Factor-7, LncTCF7), miR-410, miR-608 and so on. For instance, WSPAR has been reported to promote hepatocellular carcinoma aggressiveness through epithelial-mesenchymal transition [52]. It is worth mentioning that the IL-6 produced by non-neoplastic components within pancreatic tumors could activate proinflammatory STAT3 signaling in hepatocytes [53]. In turn, by enhancing hepatic stellate cell activation, those hepatocytes release myeloid cell chemoattractant proteins to recruit myeloid cells and increase fibrosis deposition [54]. In other words, by means of the secreted IL-6, pancreatic cancer promotes the formation of a pro-metastatic niche in the liver [55]. Thus, it would be meaningful to investigate whether WSPAR was playing essential part in the evolution of pancreatic cancer.

Currently, several clinical trials of adjuvant and neoadjuvant therapies are conducted [56], which are essential for the treatment of pancreatic cancer. As the DrugBank indicated, pivots such as Fibrinolysin and Pasireotide have already been explored in the study of pancreatic cancer [57, 58], Amcinonide came to the horizon. Amcinonide as a corticosteroid has been reported to effectively reverse the expression of the oncogene DAPK1 (death-associated protein kinase 1) in liver carcinoma [59]. While the effect of Amcinonide on pancreatic cancer have not been investigated yet, the present study offered a good insight into the further study of pancreatic cancer.

**Fig. 5:**
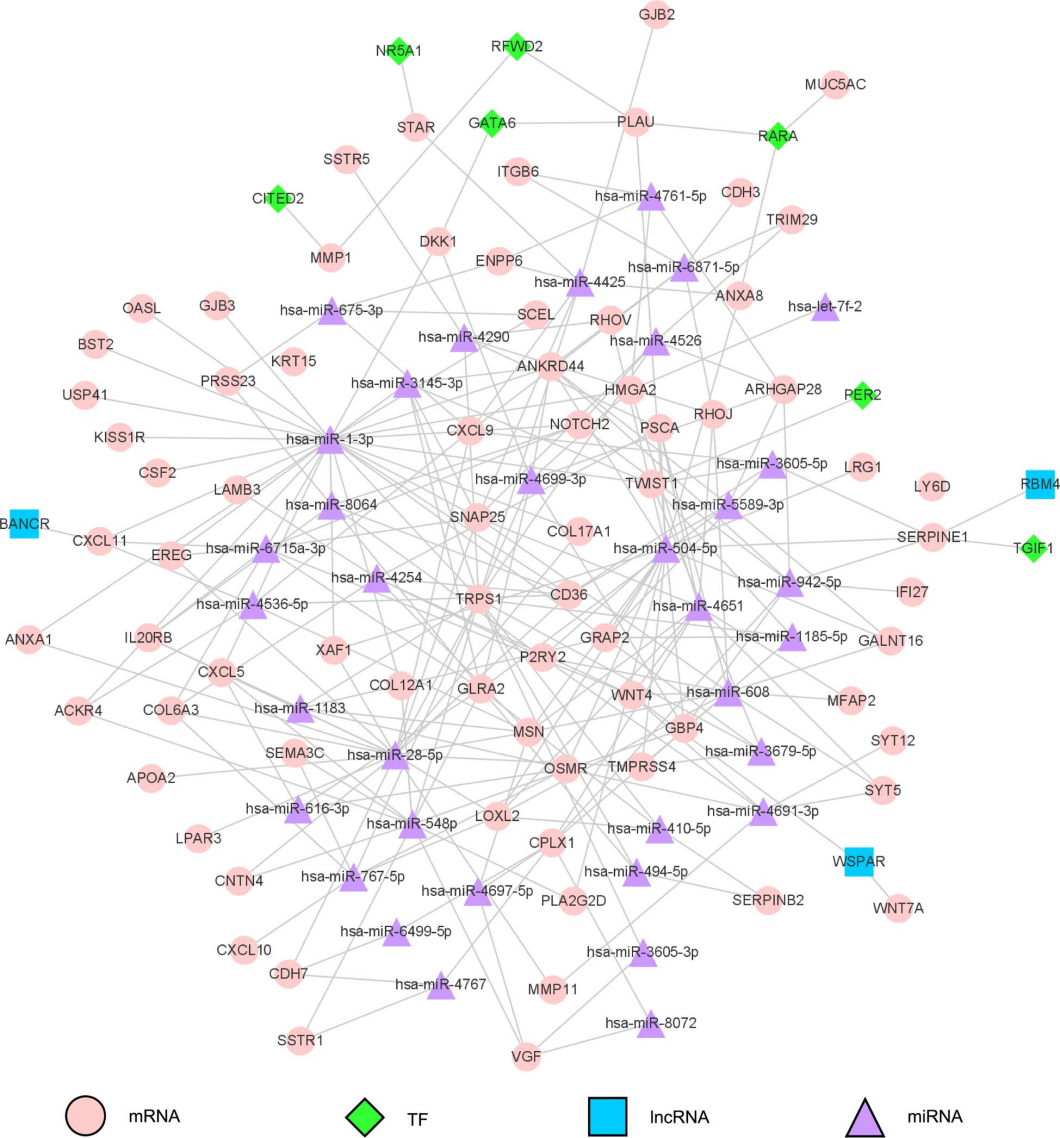
Multi-factor regulatory network on the strength of the pivot nodes Construction of multi-regulatory network after refining the results of degree and betweenness analysis with prognosis-related DEGs. A pivotal node was defined as having interactions with at least two prognosis-related genes (hypergeometric p < 0.05). There are 119 nodes in total that interact with other pivotal nodes

Pancreatic cancer evolution is essentially affected by the interaction of the intact cancer cells and the microenvironment around them, which then modifies tumor recurrence, drug resistance and prognosis of pancreatic cancer [60]. Preceding researches have done active investigation of how the tumor microenvironment was molded by the activated tumor-intrinsic genes [61]. In the present study, we provided a useful further dimension in deciphering the complicated crosstalk between tumor and its microenvironment in pancreatic cancer. Although our study sheds new light on the characteristics of the genes in microenvironment of pancreatic cancer, which in return affected tumor evolution, it still has limitations. The prognosis significance, biological functions and molecular mechanisms of the factors, including HCAR3, PPY, RFWD2, WSPAR and Amcinonide, should be investigated alone and in combination to facilitate the translational research of pancreatic cancer (Fig. 5).

## Conclusions

In summary, by means of ESTIMATE algorithm-based immune scoring and functional enrichment analysis of TCGA pancreatic cancer training cohort applied by KOBAS and GSEA, we extracted a list of genes that correlate with tumor microenvironment. These genes were subsequently tested to outline the prognosis of pancreatic cancer in an independent ICGC pancreatic cancer validation cohort. Further investigation of the new targets including DEGs, ncRNAs and TFs lead to novel insights into the potential association of tumor microenvironment with pancreatic cancer prognosis in a comprehensive manner. Drugs with potential therapeutic effects were also worth investigating. Factors such as HCAR3, PPY, RFWD2, WSPAR and Amcinonide appeared above the horizon. Last but not least, despite the grim status quo of pancreatic cancer, we still believe that light is coming [3].

## Supplementary information

**Additional file 1. Table S1.** The clinical information of pancreatic cancer patients in TCGA training cohort. **Figure S1.** ICGC cohort validation. **Figure S2.** Top 10 ncRNAs significantly associated with pancreatic cancer prognosis.**Figure S3.** The pivotal TF nodes obtained by screening the TRRUST v2 database.**Figure S4.** Correlation of RFWD2 expression with infiltration levels of CD8+ T cell, CD4+ T cell, B cell, macrophage and natural killer cell in pancreatic cancer at TIMER 2.0 database.

## Data Availability

All data generated or analysed during this study are included in this published article.

## Abbreviations

TCGA: The Cancer Genome Atlas
ESTIMATE: Estimation of Stromal and Immune cells in Malignant Tumor tissues using Expression data
DEG: Differentially expressed genes
ncRNA: Non-coding RNA
ICGC: International Cancer Genome Consortium
KOBAS: KEGG Orthology Based Annotation System
PPI: Protein–protein interaction
MCODE: Molecular Complex Detection
GSEA: Gene set enrichment analysis
TF: Transcription factor
TIMER: Tumor Immune Estimation Resource
