# Correction to: Identification of prognosis-related genes and construction of multi-regulatory networks in pancreatic cancer microenvironment by bioinformatics analysis

**DOI:** 10.1186/s12935-020-01482-7

**Published:** 2020-08-18

**Authors:** Tong Li, Qiaofei Liu, Ronghua Zhang, Quan Liao, Yupei Zhao

**Affiliations:** Department of General Surgery, Peking Union Medical College Hospital, Chinese Academy of Medical Sciences & Peking Union Medical College, Beijing, 100730 China

## Correction to: Cancer Cell Int (2020) 20:341 https://doi.org/10.1186/s12935-020-01426-1

Following publication of the original article [1], we were notified of a few errors in how the author corrections were implemented (past tense/present tense, abbreviations), which have now been fixed to ensure that the article is interpreted accurately.

The original article has been corrected.
