# Supplementary material for: Identification of prognosis-related genes and construction of multi-regulatory networks in pancreatic cancer microenvironment by bioinformatics analysis
Source: Cancer Cell Int. 2020 Jul 25;20:341. doi: 10.1186/s12935-020-01426-1 (PMC7382032; doi:10.1186/s12935-020-01426-1)
Supplement: Supplementary file 1 — Additional file 1. Table S1. The clinical information of pancreatic cancer patients in TCGA training cohort. Figure S1. ICGC cohort validation. Figure S2. Top 10 ncRNAs significantly associated with pancreatic cancer prognosis.Figure S3. The pivotal TF nodes obtained by screening the TRRUST v2 database.Figure S4. Correlation of RFWD2 expression with infiltration levels of CD8+ T cell, CD4+ T cell, B cell, macrophage and natural killer cell in pancreatic cancer at TIMER 2.0 database. [file 12935_2020_1426_MOESM1_ESM.pptx]

## Slide 1
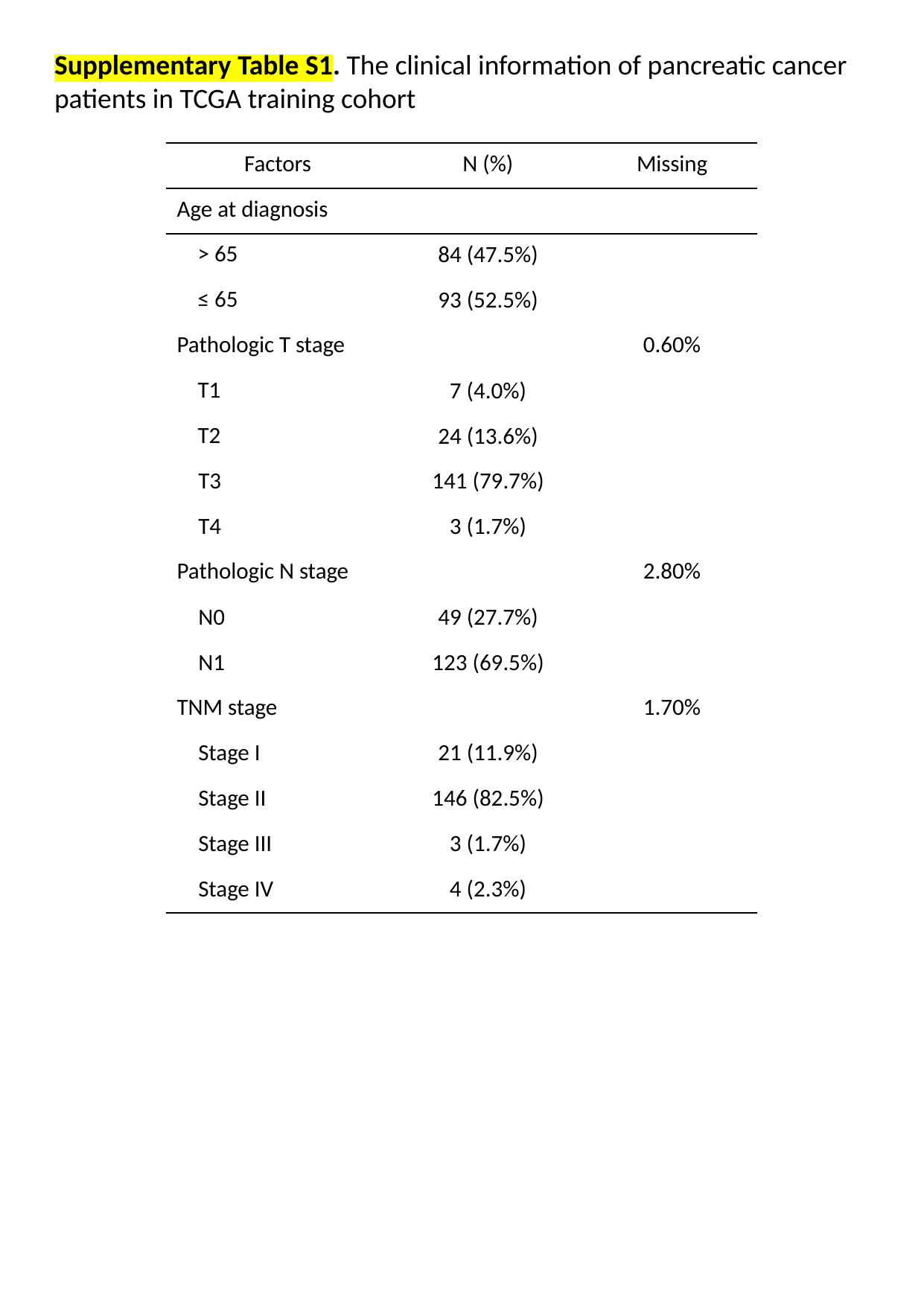

Supplementary Table S1. The clinical information of pancreatic cancer patients in TCGA training cohort
| Factors | N (%) | Missing |
| --- | --- | --- |
| Age at diagnosis | | |
| > 65 | 84 (47.5%) | |
| ≤ 65 | 93 (52.5%) | |
| Pathologic T stage | | 0.60% |
| T1 | 7 (4.0%) | |
| T2 | 24 (13.6%) | |
| T3 | 141 (79.7%) | |
| T4 | 3 (1.7%) | |
| Pathologic N stage | | 2.80% |
| N0 | 49 (27.7%) | |
| N1 | 123 (69.5%) | |
| TNM stage | | 1.70% |
| Stage I | 21 (11.9%) | |
| Stage II | 146 (82.5%) | |
| Stage III | 3 (1.7%) | |
| Stage IV | 4 (2.3%) | |

## Slide 2
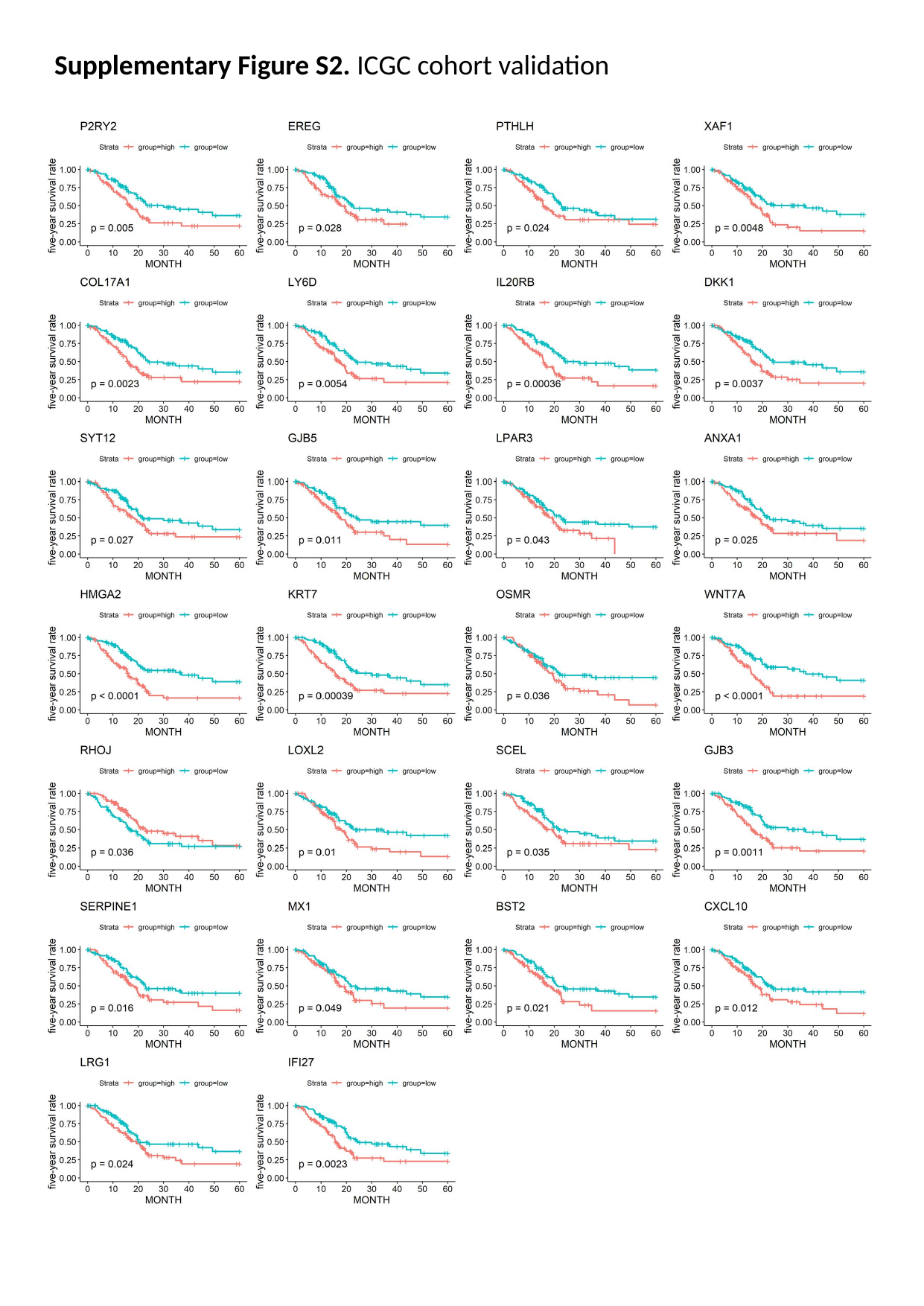

Supplementary Figure S2. ICGC cohort validation

## Slide 3
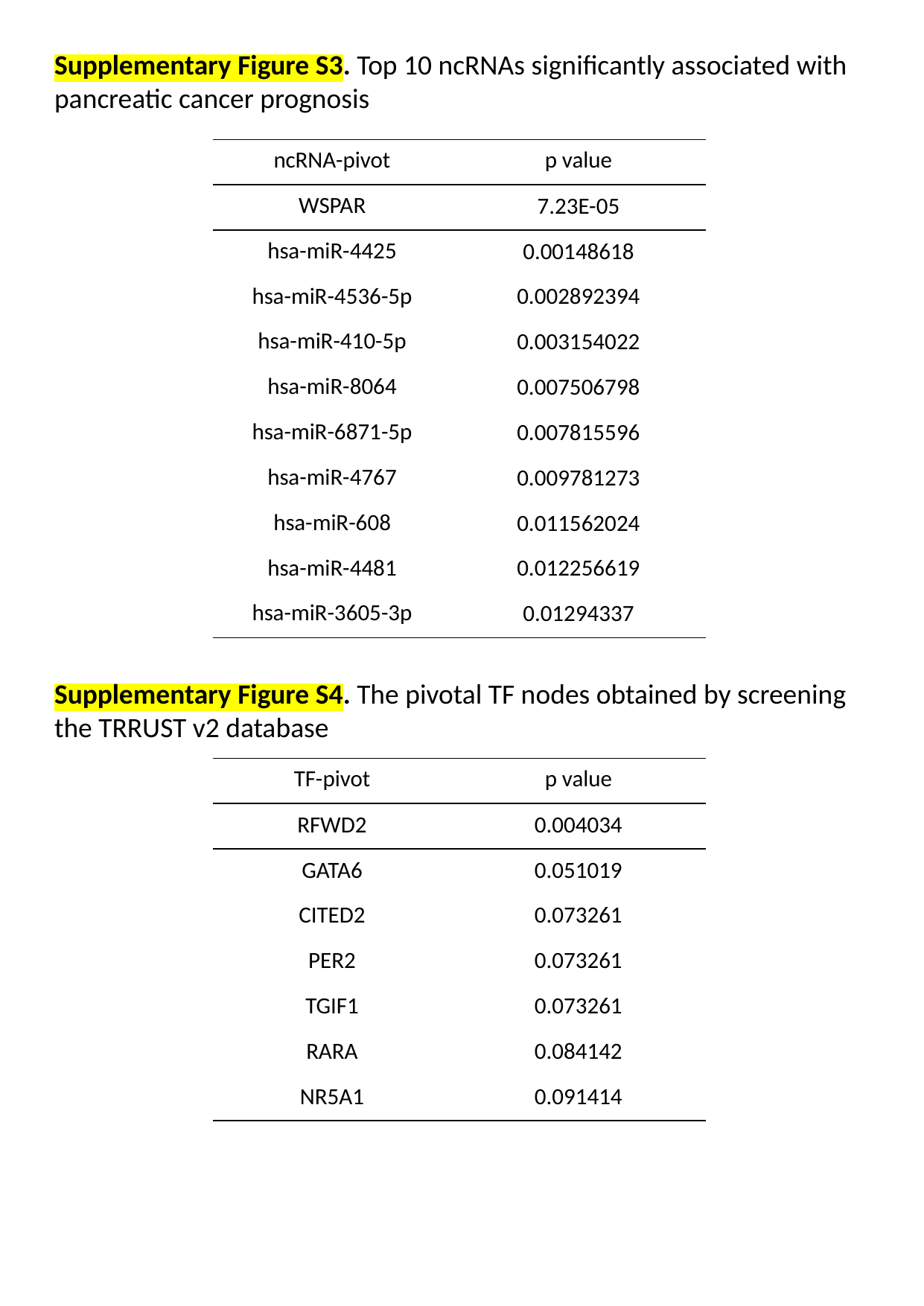

Supplementary Figure S3. Top 10 ncRNAs significantly associated with pancreatic cancer prognosis
| ncRNA-pivot | p value |
| --- | --- |
| WSPAR | 7.23E-05 |
| hsa-miR-4425 | 0.00148618 |
| hsa-miR-4536-5p | 0.002892394 |
| hsa-miR-410-5p | 0.003154022 |
| hsa-miR-8064 | 0.007506798 |
| hsa-miR-6871-5p | 0.007815596 |
| hsa-miR-4767 | 0.009781273 |
| hsa-miR-608 | 0.011562024 |
| hsa-miR-4481 | 0.012256619 |
| hsa-miR-3605-3p | 0.01294337 |
Supplementary Figure S4. The pivotal TF nodes obtained by screening the TRRUST v2 database
| TF-pivot | p value |
| --- | --- |
| RFWD2 | 0.004034 |
| GATA6 | 0.051019 |
| CITED2 | 0.073261 |
| PER2 | 0.073261 |
| TGIF1 | 0.073261 |
| RARA | 0.084142 |
| NR5A1 | 0.091414 |

## Slide 4
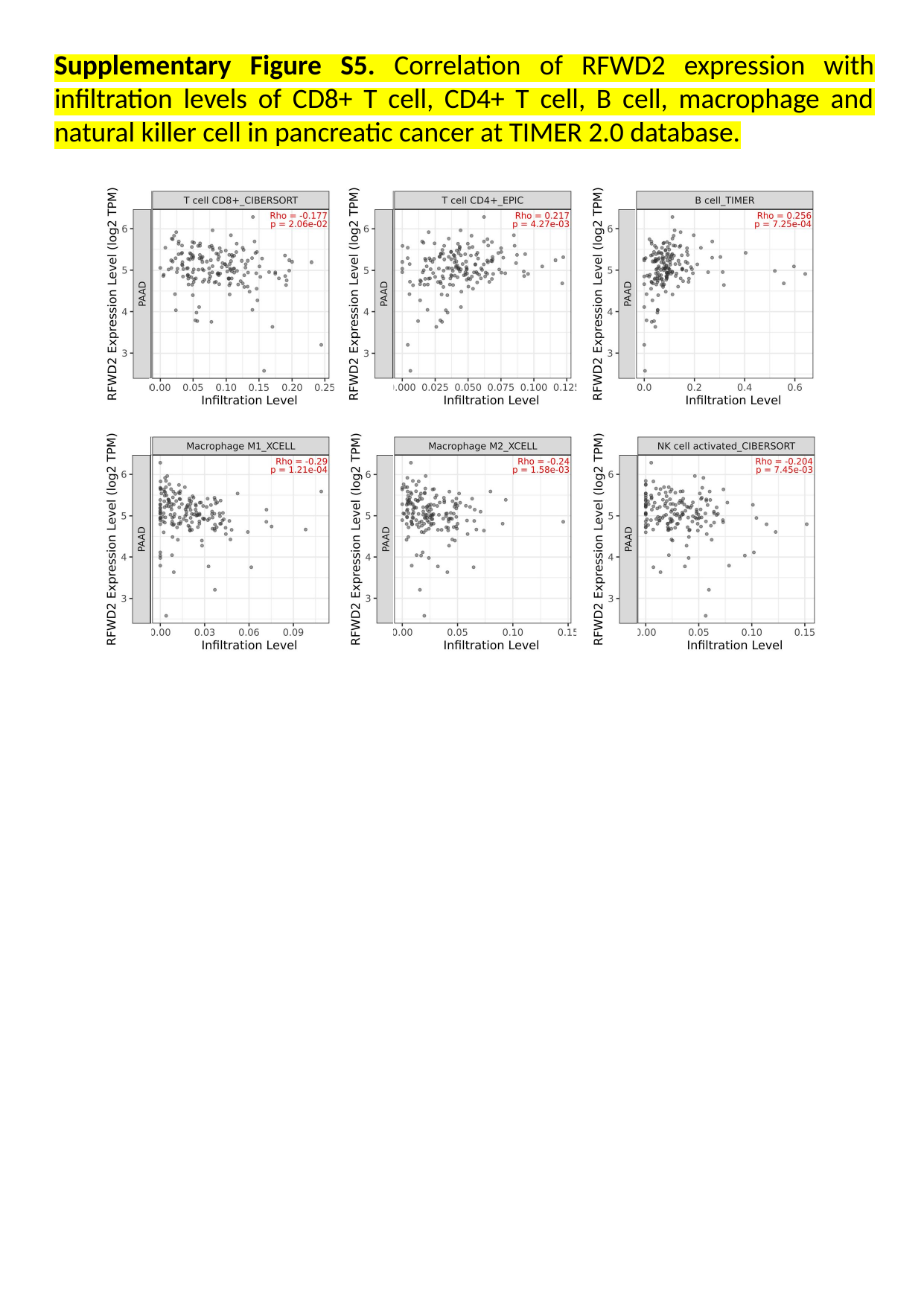

Supplementary Figure S5. Correlation of RFWD2 expression with infiltration levels of CD8+ T cell, CD4+ T cell, B cell, macrophage and natural killer cell in pancreatic cancer at TIMER 2.0 database.
